# A Multifunctional (-)-Meptazinol-Serotonin Hybrid Ameliorates Oxidative Stress-Associated Apoptotic Neuronal Death and Memory Deficits via Activating the Nrf2/Antioxidant Enzyme Pathway

**DOI:** 10.1155/2023/6935947

**Published:** 2023-02-09

**Authors:** Faxue Zhao, Lin Zhao, Yan Zhou, Xiaofang Tan, Yang Yang, Wenwen Ni, Wei Zheng, Hongzhuan Chen, Yu Qiu, Juan Li

**Affiliations:** ^1^Department of Pharmacology and Chemical Biology, Shanghai Jiao Tong University School of Medicine, 280 South Chongqing Road, Shanghai 200025, China; ^2^NHC Key Laboratory of Reproduction Regulation, Shanghai Institute for Biomedical and Pharmaceutical Technologies, 2140 Xietu Road, Shanghai 200032, China; ^3^Institute of Interdisciplinary Integrative Biomedical Research, Shanghai University of Traditional Chinese Medicine, 1200 Cailun Road, Shanghai 201210, China

## Abstract

The pathogenesis of Alzheimer's disease (AD) involves multiple pathophysiological processes. Oxidative stress is a major cause of AD-associated neuronal injury. The current research was designed to examine whether a novel (-)-meptazinol-serotonin hybrid (Mep-S) with potent antioxidant activity and additional inhibitory properties for acetylcholinesterase (AChE) activity could attenuate oxidative neuronal damage and cognitive deficits. In human SH-SY5Y cells, Mep-S suppressed H_2_O_2_-induced apoptosis by restoring mitochondrial membrane potential and inhibiting caspase-3 activation. Meanwhile, it attenuated oxidative stress elicited by H_2_O_2_ through lessening generation of reactive oxygen species as well as enhancing production of glutathione (GSH) and activity of superoxide dismutase (SOD). Mechanistically, Mep-S promoted nuclear translocation of a transcription factor nuclear factor E2-related factor-2 (Nrf2) in H_2_O_2_-challenged cells. This effect was accompanied by reduction in Kelch-like ECH-associated protein-1 (Keap1) levels as well as augmentation of Akt phosphorylation and expression of heme oxygenase-1 (HO-1) and NAD(P)H quinine oxidoreductase-1 (NQO-1). Molecular docking analysis revealed that Mep-S may disrupt the protein-protein interactions between Keap1 and Nrf2. In an *in vivo* mouse model, Mep-S attenuated scopolamine-caused cognitive deficits with inhibition of apoptotic neuronal death and brain AChE activity. Furthermore, the scopolamine-induced impairment of total antioxidant capacity and reduction in SOD1, SOD2, and *γ*-glutamate-cysteine ligase expression in the brain were counteracted by Mep-S, accompanied by decreased Keap1 levels, increased Akt catalytic subunit and Nrf2 phosphorylation, and decreased Nrf2, HO-1, and NQO-1 expression. Collectively, our results suggest that Mep-S ameliorates apoptotic neuronal death and memory dysfunction associated with oxidative stress by regulating the Nrf2/antioxidant enzyme pathway through inactivating Keap1 and phosphorylating Nrf2 via Akt activation. Therefore, Mep-S may be a potential lead for multitarget neuroprotective agents to treat AD-like symptoms.

## 1. Introduction

Alzheimer's disease (AD), predominantly affecting the elderly people and causing progressive cognitive decline, involves a multifactorial pathogenesis. The primary neuropathological hallmarks in AD brains include the presence of extracellular amyloid-*β* (A*β*) deposition as senile plaques, intracellular accumulation of hyperphosphorylated tau as neurofibrillary tangles, and loss of cholinergic neurons [1]. Currently, no disease-modifying treatment has been established. To date, FDA-approved drugs for AD therapy include inhibitors of acetylcholinesterase (AChE) or N-methyl-D-aspartate receptors [2–4]. Although benefiting for cognitive symptoms, they fail to prevent pathological processes of the disease [1, 5]. Notably, the A*β*-directed antibody aducanumab received accelerated approval from the FDA in 2021; however, its efficacy for AD is controversial [6]. Since the increasing AD population may pose a significant burden on family and public health, it is urgent to develop innovative disease-modifying medications for this disease.

AD pathogenesis involves multiple pathological processes that are closely linked and interact [1]. Substantial evidence shows that oxidative stress, caused by reactive oxygen species (ROS) overproduction, is an early and sustained event in AD brain [7–9]. In addition to directly causing neuronal damage (particularly in cognition-related areas, e.g., the hippocampus), oxidative stress may aggravate A*β* and tau pathology [9, 10]. Therefore, oxidative neuronal damage is considered a major factor associated with both the onset and progression of AD pathogenesis. Evidence shows that agents with antioxidant activity could benefit for AD therapy [11–13].

**Figure 1:**
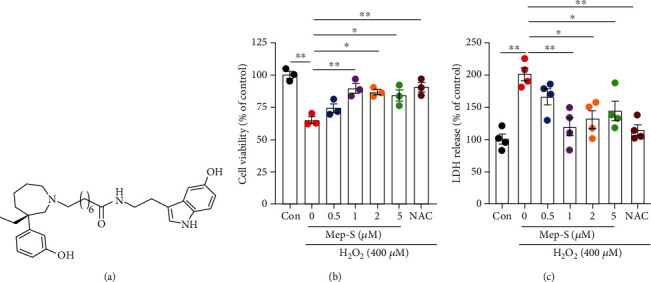
Mep-S protects against H_2_O_2_-induced neurotoxicity in SH-SY5Y cells. (a) Chemical structure of Mep-S. Mep-S protected against H_2_O_2_-induced cell death (b) and LDH release (c) in SH-SY5Y cells. The cells were pretreated with Mep-S (0.5-5 *μ*M) or N-acetyl-L-cysteine (NAC, 1 mM) for 1 h prior to stimulation with H_2_O_2_ (400 *μ*M) for 24 h. Data are presented as the mean ± SEM of 3-4 independent experiments. ^∗^*P* < 0.05 and ^∗∗^*P* < 0.01.

The multifactorial nature of AD warrants multitarget intervention for the disease. To this end, the exploration of multi-target-directed ligands (MTDLs) has become a hotspot in the development of new therapies for AD [14–17]. Considering the critical roles of oxidative stress in AD pathogenesis, MTDLs with antioxidant potency may be favorable for AD therapy. In fact, the development of anti-AD MTDLs with antioxidant activity has recently attracted much attention [15, 18]. In a previous research, we reported a number of (-)-meptazinol-melatonin/serotonin hybrids with multiple pharmacological properties [19]. The results of *in vitro* testing demonstrate that a novel (-)-meptazinol-serotonin hybrid, Mep-S (Figure 1(a)), in addition to inhibiting AChE activity, exhibits high antioxidant potency (7.2-fold of Trolox). Additionally, it displays a favorable effect on neuronal cell viability and shows sufficient blood-brain barrier penetrability. Thus, this compound could serve as a potential MTDL lead for AD therapy.

In this work, we examined whether Mep-S could ameliorate oxidative neuronal injury by *in vitro* and *in vivo* testing. The results showed that Mep-S protects against H_2_O_2_-induced neuronal apoptosis and ameliorates scopolamine-caused cognitive deficits. Further mechanistic studies revealed that the nuclear factor E2-related factor-2 (Nrf2)/antioxidant enzyme pathway was associated with the neuroprotective effect of Mep-S.

## 2. Materials and Methods

### 2.1. Reagents

Mep-S was synthesized in our laboratory as previously described [19]. Scopolamine, H_2_O_2_, and N-acetylcysteine (NAC) were the products of Sigma-Aldrich (St. Louis, MO, USA). Dulbecco's modified Eagle's medium (DMEM)/F-12 (1 : 1), phosphate-buffered saline (PBS), and a mixture of penicillin and streptomycin were the products of Gibco (Grand Island, NY, USA). The rabbit-derived antibodies against NAD(P)H quinine oxidoreductase-1 (NQO-1; cat #ab80588), heme oxygenase-1 (HO-1), Kelch-like ECH-associated protein-1 (Keap1), Nrf2, phospho-Nrf2 (S40), and B-cell lymphoma 2 (Bcl-2) were the products of Abcam (Cambridge, MA, USA). The rabbit-derived antibodies against caspase-3 (D3R6Y), phospho-Akt (Ser473), Akt, and Bax were the products of Cell Signaling Technology (CST; Danvers, MA, USA). Mep-S was dissolved in dimethyl sulfoxide (DMSO; Sigma), and NAC was dissolved in normal saline to obtain stock solutions (20 mg/ml), which were diluted with saline (for *in vivo* experiments) or culture medium (for *in vitro* tests). The Mep-S working solutions contain less than 2.5% of DMSO.

### 2.2. Animals

ICR mice (male, weighing 25–30 g; Shanghai Laboratory Animal Center, Chinese Academy of Science, Shanghai, China) were kept under a light/dark cycle of 12 h/12 h in a room with constant temperature, with free access to water and food. The experimental procedures were performed following principles of the Declaration of Helsinki. The Ethics Committee of Shanghai Jiao Tong University School of Medicine approved them.

### 2.3. Cell Culture

SH-SY5Y human neuroblastoma cells were cultured in a DMEM/F-12 mixture containing fetal bovine serum (FBS, 10%; Sigma), L-glutamine (2 mM; Gibco), penicillin (100 U/ml), and streptomycin (100 *μ*g/ml) at 37°C. They were used for experiments after growing to the required confluence, with culture medium changed to serum-free medium prior to addition of drugs.

### 2.4. Detection of Cell Viability

Cell viability was assessed with a Cell Counting Kit-8 (CCK-8; Dojindo, Kumamoto, Japan). After being seeded in 96-well plates at 2 × 10^4^ cells per well and growing to the required confluence, SH-SY5Y cells received 24 h exposure to H_2_O_2_ with or without preincubation with Mep-S or NAC (serving as a positive control of antioxidants) for 1 h. Thereafter, the cells were incubated with CCK-8 (20 *μ*l, 5 mg/ml) at 37°C for 2 h. Cell viability, which was evaluated based on the absorbance measured with a test wavelength (570 nm) and a reference wavelength (655 nm), was normalized to the percentage of control group.

### 2.5. Detection of Lactate Dehydrogenase (LDH) Release

The cell-released LDH content was detected using an LDH cytotoxicity assay kit (Beyotime Institute of Biotechnology, Shanghai, China) following instructions of the manufacturer. In brief, the cells were subjected to 24 h stimulation with H_2_O_2_ with or without preincubation with Mep-S or NAC. Then, the culture medium (120 *μ*l) was aspirated to mix with LDH test working solution (60 *μ*l). The absorbance was detected at a wavelength of 490 nm after 30 min incubation at room temperature.

### 2.6. Annexin V/PI Staining

Neuronal apoptosis was measured by flow cytometry using a commercial FITC Annexin V Apoptosis Detection Kit (BD Pharmingen, Franklin Lakes, NJ, USA). In brief, the cells, which were seeded in 6-well plates at 5 × 10^5^ cells per well, were stimulated with H_2_O_2_ for 24 h with or without preincubation with Mep-S or NAC. Afterwards, they were harvested with trypsin-ethylenediaminetetraacetic acid (EDTA, 0.25%; Gibco) and centrifugated at 1500 rpm for 5 min at 4°C. Thereafter, cell pellets were resuspended in FITC Annexin V- and propidium iodide-containing binding buffer, followed by 15 min incubation at room temperature and subsequent detection on a flow cytometer (Thermo Fisher Scientific, Rockford, IL, USA).

### 2.7. Mitochondrial Membrane Potential (MMP) Detection

MMP was evaluated with JC-1 fluorescence dye. It exhibits potential-dependent accumulation in mitochondria, where it forms J-aggregates and exhibits red fluorescence. Decreased MMP favors JC-1 monomers that yield green fluorescence. Briefly, the cells were challenged with H_2_O_2_ for 24 h with or without preincubation with Mep-S or NAC. Afterwards, they were centrifuged for 5 min at 1500 rpm at 4°C after 20 min incubation with JC-1 dye (2 *μ*M; Beyotime) at 37°C. The cell pellets were resuspended in JC-1 staining buffer and detected on a flow cytometer.

### 2.8. Measurement of Intracellular ROS Accumulation

The intracellular ROS was detected with 2′7′-dichlorofluorescein-diacetate (DCFH-DA; Sigma), a fluorescent dye. After deacetylation, it reacts with intracellular radicals (predominantly generated by hydrogen peroxide) and is transformed into DCF, an intracellularly retained fluorescent product. The cells were challenged with H_2_O_2_ for 2 h with or without preincubation with Mep-S or NAC. Afterwards, they were probed with DCFH-DA (10 *μ*M; Sigma) at 37°C for 30 min. The fluorescence intensity (Ex 488 nm/Em 530 nm) was examined under a confocal microscope (Leica TCS SP2 AOBS; Leica, Wetzlar, Germany).

### 2.9. Detection of Glutathione (GSH) Content and Superoxide Dismutase (SOD) Activity

A GSH assay kit (Abbkine Scientific Co., Ltd, Wuhan, China) and SOD assay kit (Beyotime) were applied for detection of GSH content and SOD activity, respectively. The cells received 24 h H_2_O_2_ stimulation with or without preincubation with Mep-S or NAC. Afterwards, the supernatants of cell homogenates were collected and used for assay of GSH content and SOD activity.

### 2.10. Molecular Docking

Molecular docking simulation was conducted to explore the potential impact of Mep-S on Keap1-Nrf2 protein-protein interactions (PPI). The X-ray crystal structure of mouse Keap1 Kelch domain (at 1.21 Å resolution), which serves as the recognition module for Nrf2 [20, 21], was acquired from the Protein Data Bank (code: 6ZF4). Water molecules and heteroatoms in protein structures were discarded before addition of hydrogen atoms. The 3D coordinates of Mep-S (serving as the ligand) were obtained using CORINA Version 3.0 (Molecular Networks GmbH, Erlangen, Germany). After acquirement of the final ligand conformations by energy minimization with Tripos force field, molecular docking simulation was conducted with AutoDock Vina 1.1.2 to obtain an ensemble of docked conformations. The best conformation was chosen according to the lowest docked energy.

### 2.11. Design of Animal Experiments

Mice were randomized into 6 groups (*n* = 10). The control animals were given intraperitoneal (i.p.) injection of saline per day while the scopolamine-treated animals were daily challenged with scopolamine (2 mg/kg) 15 min before the behavioral test. The scopolamine+Mep-S-treated groups received a daily injection of Mep-S at 0.5, 1, 2, and 5 mg/kg, respectively, 15 min prior to the delivery of scopolamine. On days 1-6, the Morris water maze test was performed for behavioral assessment. In a separate experiment, 4 groups of mice received a daily injection of saline and Mep-S (at 0.5, 2, and 5 mg/kg), respectively, prior to the injection of scopolamine for 6 days. The control group received daily saline injection. The mice were transcardially perfused with saline after euthanasia 24 h after the last drug administration. After removal of the brain, one hemisphere was immediately postfixed for 2 h in 10% formalin solution and then subjected to paraffin embedding and sectioning for histological analyses. The other hemisphere was immediately stored at -80°C for subsequent quantitative real-time polymerase chain reaction (qPCR), western blot, and biochemical analyses.

### 2.12. Morris Water Maze Test

The Morris water maze test was performed as previously described [22]. Briefly, mice were placed in the experimental room to adapt to the environment 24 h before the testing. The testing was conducted in a black circular tank with a diameter of 140 cm, which contained water (22 ± 1.0°C) to a 30 cm depth. During the 5-day acquisition testing, each mouse received 4 trials daily. The interval between trials was 30 s. The test animals that were able to find the submerged platform (9 cm diameter, located on the center of a fixed quadrant) within 60 s were allowed to stay for 30 s on it. Animals failing to find the platform within 60 s were guided to stay for 30 s on it. During the probe test on day 6, retention of spatial memory was assessed, with the platform being removed. The test animals were allowed to swim for 60 s. Swimming traces were analyzed to evaluate spatial learning capacity.

### 2.13. Western Blot

SH-SY5Y cells as well as mouse hippocampi and cerebral cortex were lysed in radio-immunoprecipitation assay (RIPA) buffer containing protease inhibitor cocktail (1%) and phenylmethanesulfonyl fluoride (PMSF, 1%) protease inhibitor, followed by 10 min centrifugation at 15,000 rpm at 4°C. For cellular nuclear and cytoplasmic fractions, a commercial CelLytic NuCLEAR Extraction Kit (Sigma) was used to separate cellular cytoplasmic and nuclear proteins. Protein samples were separated on 10% sodium dodecyl sulfate-polyacrylamide gel. Thereafter, they were transferred to a polyvinylidene fluoride (PVDF) membrane (Merck Millipore, Tullagreen, Ireland), followed by blockade with 5% nonfat milk. Afterwards, the membrane was subjected to overnight incubation at 4°C with primary antibodies (1 : 1000) against caspase-3, HO-1, NQO-1, Keap1, phospho-Akt, Akt, Nrf2, phospho-Nrf2, Bcl-2, Bax, *β*-actin (CST), glyceraldehyde 3-phosphate dehydrogenase (GAPDH; Abcam), or H3 (Abcam), followed by 1 h incubation at room temperature with a diluted (1 : 10000) horseradish peroxidase-conjugated antibody. After visualization with an enhanced chemiluminescence (Pierce, Rockford, IL, USA), the bands were analyzed and quantified.

### 2.14. qPCR

After extraction with a commercial RNA Extraction Kit (Takara, Otsu, Shiga, Japan), 1 *μ*g of RNA extracted from mouse brain tissues was reverse-transcribed to cDNA. qPCR was conducted on LightCycler 480 Instrument II (Roche, Indianapolis, IN, USA) with a Takara SYBR Premix Ex Taq Kit. The following are primer sequences used: SOD1, 5′-GTGATTGGGATTGCGCAGTA-3′ (forward) and 5′-TGGTTTGAGGGTAGCAGATGAGT-3′ (reverse); SOD2, 5′-TTAACGCGCAGATCATGCA-3′ (forward) and 5′-GGTGGCGTTGAGATTGTTCA-3′ (reverse); *γ*-glutamate-cysteine ligase catalytic subunit (GCLC), 5′-AGCACAGGGTGACAGAAGAG-3′ (forward) and 5′-GAGGGACTCTGGTCTTTGTG-3′ (reverse); and GAPDH, 5′-AACGACCCCTTCATTGAC-3′ (forward) and 5′-TCCACGACATACTCAGCAC-3′ (reverse). Target gene expression was evaluated with the 2^-*ΔΔ*ct^ method.

### 2.15. Hematoxylin and Eosin (H&E) Staining

Serial coronal sections of mouse brains were deparaffinized and rehydrated. After hematoxylin and eosin staining, the histopathological properties were evaluated under a Leica DFC 320 digital camera.

### 2.16. Immunofluorescence Staining

Serial coronal sections of mouse brains were incubated at 4°C overnight with an antibody specific for Nrf2 (1 : 100), followed by 1 h incubation at room temperature with a Alexa Fluor 488-conjugated antibody (Invitrogen, Carlsbad, CA, USA). The nucleus was stained with 4′,6-diamidino-2-phenylindole (DAPI). The fluorescent intensity was evaluated under a Leica confocal microscope.

### 2.17. Detection of AChE Activity

Mouse brain samples were subjected to homogenate and 10 min centrifugation (2500 rpm) at 4°C. After addition of PBS (0.05 M, pH 7.2) containing 0.25 mM 5,5′-dithiobis-(2-nitrobenzoic) acid (DTNB; Sigma), the supernatants were added with 1 mM acetylthiocholine (Sigma). The absorbance change at the wavelength of 412 nm was detected using a Varioskan Flash multimode reader (Thermo Fisher). AChE activity was presented as units/mg protein.

### 2.18. Detection of Total Antioxidant Capacity

Mouse brain samples were homogenized and centrifuged at 12,000 rpm for 5 min at 4°C. Total antioxidant capacity was detected with a commercial kit (Beyotime) following the instructions of the manufacturer.

### 2.19. Statistical Analysis

Experimental data were presented as the mean ± standard error of the mean (SEM) and statistically analyzed with analysis of variance (ANOVA; one-way or two-way) and Bonferroni's posttest. A *P* value of less than 0.05 was regarded as statistically significant.

## 3. Results

### 3.1. Protection of Mep-S against H_2_O_2_-Evoked Cytotoxicity

We previously found potent antioxidant activity of Mep-S (7.2-fold of Trolox) in a cell-free system [19]. In this work, we further examined the antioxidant effect of this compound in human SH-SY5Y neuronal cells stimulated with H_2_O_2_. We first detected the effect of Mep-S on viability of neuronal cells. The results showed that Mep-S did not influence the survival of SH-SY5Y cells at concentrations of 1-20 *μ*M (one-way ANOVA, *F*(5, 18) = 0.25, *P* = 0.93; Supplementary material, Figure S1A). We then treated the cells with H_2_O_2_ and found that H_2_O_2_ induced dose-dependent cell death at concentrations of 50-800 *μ*M (one-way ANOVA, *F*(5, 12) = 59.81, *P* < 0.0001; Supplementary material, Figure S1B). A dose of 400 *μ*M H_2_O_2_ was adopted in the subsequent *in vitro* testing.

To detect whether Mep-S could inhibit H_2_O_2_-induced neurotoxicity, the cells received 1 h pretreatment with Mep-S (0.5-5 *μ*M) before H_2_O_2_ stimulation. As demonstrated in Figure 1(b), cell viability was obviously different between groups (one-way ANOVA, *F*(6, 14) = 11.65, *P* < 0.0001). The viability of cells incubated with H_2_O_2_ (65.20 ± 2.64%) was higher than control group (*P* < 0.01). The survival rate was markedly increased to 89.79 ± 3.90%, 86.91 ± 1.98%, and 84.20 ± 4.49% in cells pretreated with Mep-S at 1, 2, and 5 *μ*M, respectively (*P* < 0.05 or *P* < 0.01 vs. H_2_O_2_-treated cells). Similarly, survival of cells receiving NAC (1 mM; as a positive control) pretreatment (90.72 ± 3.65%) was overtly elevated compared with H_2_O_2_-treated cells (*P* < 0.01). In addition, LDH release was obviously different between groups (one-way ANOVA, *F*(6, 21) = 8.46, *P* < 0.0001; Figure 1(c)). Mep-S at doses of 1, 2, and 5 *μ*M significantly suppressed H_2_O_2_-evoked LDH release (*P* < 0.01 at 1 *μ*M and *P* < 0.05 at 2 or 5 *μ*M vs. H_2_O_2_-treated group). LDH release from cells pretreated with NAC was also significantly lower than that from H_2_O_2_-challenged cells (*P* < 0.01).

### 3.2. Protection of Mep-S against H_2_O_2_-Evoked Apoptotic Neuronal Death

**Figure 2:**
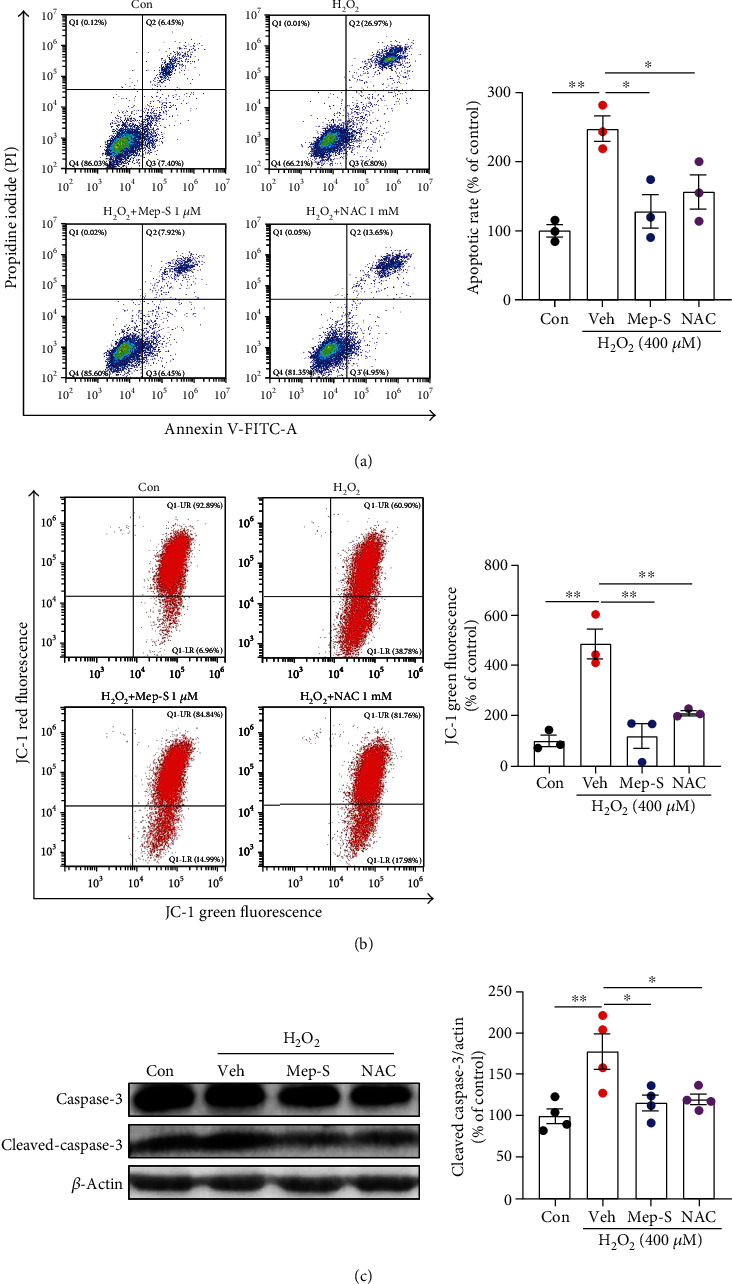
Mep-S protects against H_2_O_2_-induced neuronal apoptosis in SH-SY5Y cells. Mep-S inhibited H_2_O_2_-induced apoptosis (a) and loss of mitochondrial membrane potential (b) in SH-SY5Y cells. (c) Mep-S inhibited H_2_O_2_-induced upregulation of cleaved caspase-3 levels. The cells were pretreated with Mep-S (1 *μ*M) or NAC (1 mM) for 1 h and then exposed to H_2_O_2_ (400 *μ*M) for another 24 h. Data are presented as the mean ± SEM of 2-4 independent experiments. ^∗^*P* < 0.05 and ^∗∗^*P* < 0.01.

We then examined neuronal apoptosis to further explore the protective effect of Mep-S (at 1 *μ*M, the concentration at which Mep-S showed the most obvious effect) on H_2_O_2_-challenged SH-SY5Y cells. Based on the results of Annexin V/PI staining in SH-SY5Y cells (one-way ANOVA, *F*(3, 8) = 10.17, *P* = 0.0042; Figure 2(a)), the apoptotic rate of cells challenged with H_2_O_2_ increased by 1.47-fold relative to control group (*P* < 0.01). Pretreatment with Mep-S or NAC markedly decreased the cellular apoptotic rate compared with H_2_O_2_-treated group (*P* < 0.05).

Dissipation of MMP is closely associated with the initiation of the intrinsic pathway of apoptosis and, thus, is regarded as a marker of apoptotic cells [23]. As shown in Figure 2(b) (one-way ANOVA, *F*(3, 8) = 19.40, *P* = 0.0005), the emission of JC-1 in H_2_O_2_-treated SH-SY5Y cells was shifted from red to green (indicating a loss of MMP and depolarization of the mitochondrial membrane), with an increased intensity of JC-1 green fluorescence compared to H_2_O_2_-treated cells (*P* < 0.01). Mep-S or NAC pretreatment markedly decreased the intensity of JC-1 green fluorescence (*P* < 0.01 vs. H_2_O_2_-challenged group). Activation of caspase-3 is a downstream event of MMP collapse [23]. As shown in Figure 2(c) (one-way ANOVA, *F*(3, 12) = 6.75, *P* = 0.0064), H_2_O_2_ markedly elevated cleaved caspase-3 level to 177.3 ± 21.5% relative to control values (*P* < 0.01). Mep-S or NAC pretreatment significantly repressed the upregulation of cleaved caspase-3 levels compared to H_2_O_2_-challenged group (*P* < 0.05).

### 3.3. Mep-S Inhibits H_2_O_2_-Induced ROS Production along with Decrease in GSH Content and SOD Activity

**Figure 3:**
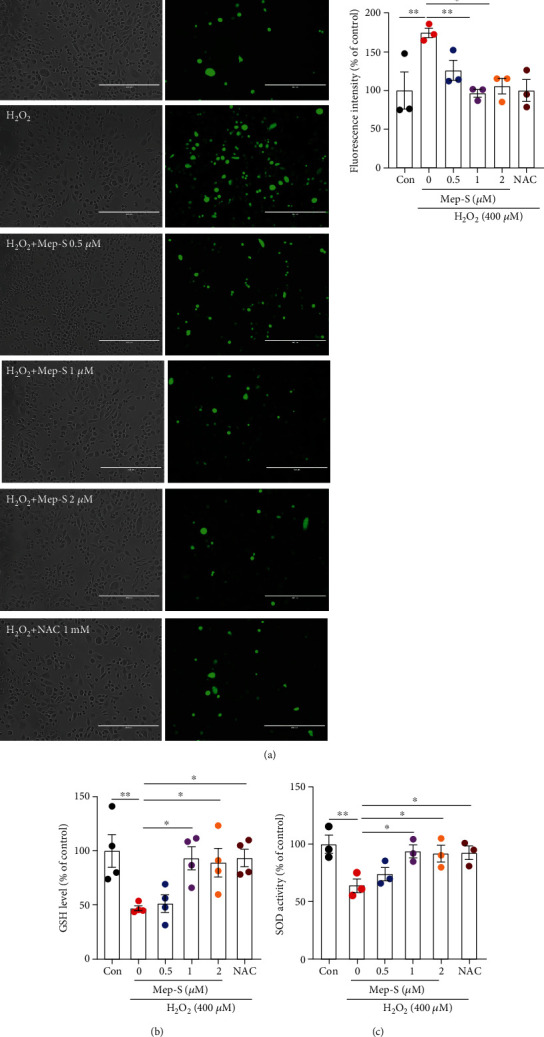
Mep-S inhibits H_2_O_2_-induced ROS generation and decreases GSH content and SOD activity in SH-SY5Y cells. (a) Mep-S inhibited H_2_O_2_-induced intracellular ROS accumulation, as demonstrated by decreased DCFH-DA fluorescence intensity. Scale bar = 400 *μ*m. Mep-S inhibited the H_2_O_2_-induced decrease in GSH content (b) and SOD activity (c). The cells were pretreated with Mep-S (0.5-2 *μ*M) or NAC (1 mM) for 1 h prior to stimulation with H_2_O_2_ (400 *μ*M) for 2 h (a) or 24 h (b, c). Data are presented as the mean ± SEM of 2-4 independent experiments. ^∗^*P* < 0.05 and ^∗∗^*P* < 0.01.

It was shown based on one-way ANOVA that intracellular ROS level is obviously different between groups (*F*(5, 12) = 4.89, *P* = 0.011; Figure 3(a)). Stimulation with H_2_O_2_ increased ROS levels within SH-SY5Y cells, with significantly higher DCHF-DA fluorescence intensity compared to control cells (*P* < 0.01). Pretreatment with Mep-S (1 and 2 *μ*M) or NAC markedly inhibited intracellular ROS accumulation (*P* < 0.05 or *P* < 0.01 vs. H_2_O_2_-treated cells) (Figure 3(a)). In addition, one-way ANOVA showed significantly different GSH content (*F*(5, 18) = 5.06, *P* = 0.0045; Figure 3(b)) and SOD activity (*F*(5, 12) = 4.61, *P* = 0.014; Figure 3(c)) among groups. The H_2_O_2_-challenged cells showed significantly decreased GSH content and SOD activity compared to control group (*P* < 0.01). Mep-S (1 and 2 *μ*M) or NAC pretreatment significantly increased GSH content and SOD activity compared to H_2_O_2_-treated group (*P* < 0.05).

### 3.4. Mep-S Activates the Nrf2/Antioxidant Enzyme Signaling Axis in H_2_O_2_-Challenged Cells

**Figure 4:**
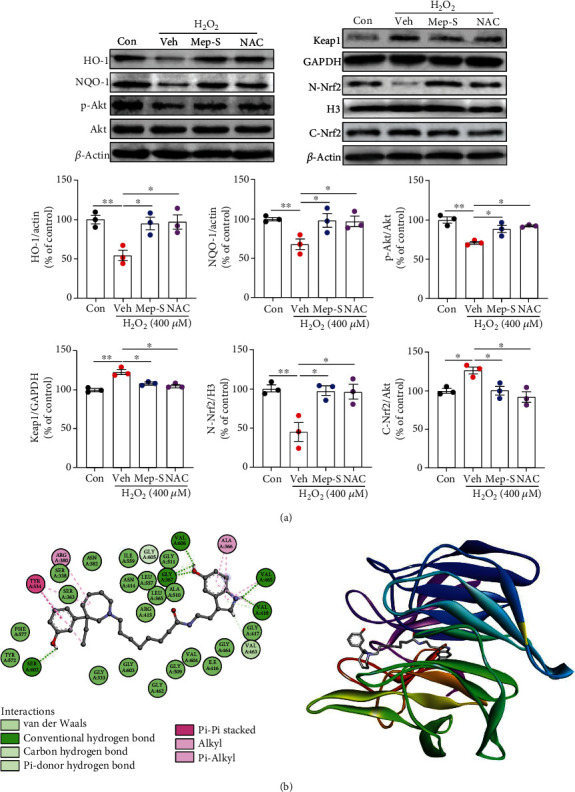
Effect of Mep-S on the Nrf2/antioxidant enzyme pathway in H_2_O_2_-stimulated SH-SY5Y cells. (a) Protein levels of HO-1, NQO-1, Keap1, Akt, phosphorylated Akt, and nuclear and cytoplasmic Nrf2 in SH-SY5Y cells detected by western blot analysis. The cells were pretreated with Mep-S (1 *μ*M) or NAC (1 mM) for 1 h prior to stimulation with H_2_O_2_ (400 *μ*M) for 24 h. Data are presented as the mean ± SEM of 3 independent experiments. One-way ANOVA: *F*(3, 8) = 8.34, *P* = 0.0076 (HO-1); *F*(3, 8) = 5.35, *P* = 0.026 (NQO-1); *F*(3, 8) = 14.07, *P* = 0.0015(p-Akt); *F*(3, 8) = 21.24, *P* = 0.0004 (Keap1); *F*(3, 8) = 9.83, *P* = 0.0046 (N-Nrf2); *F*(3, 8) = 8.35, *P* = 0.0076 (C-Nrf2). ^∗^*P* < 0.05 and ^∗∗^*P* < 0.01. (b) Interactions (left) and docking representation (right) of Mep-S with the Kelch domain of mouse Keap1 (PDB code: 6ZF4). Mep-S is shown as a gray stick model, and Keap1 is shown as a cartoon. In the 2D diagram (the left panel), key residues of Keap1 are represented as circular shapes and colored according to the interaction types.

Evidence shows that Keap1/Nrf2 axis is crucial for protection of neurons against oxidative injury by inducing antioxidant enzyme (e.g., HO-1 and NQO-1) expression to suppress the intracellular ROS level [24]. We therefore explored the effect of Mep-S on this pathway. As shown in Figure 4(a), HO-1 and NQO-1 levels in H_2_O_2_-challenged SH-SY5Y cells overtly declined (*P* < 0.01 vs. control group). Concurrently, Keap1 (a negative Nrf2 modulator) and cytoplasmic Nrf2 levels increased, while nuclear Nrf2 level decreased after H_2_O_2_ stimulation compared with control group (*P* < 0.05 or *P* <0.01). Mep-S or NAC pretreatment markedly inhibited the H_2_O_2_-induced reduction in HO-1, NQO-1, and nuclear Nrf2 levels as well as elevation of Keap1 and cytoplasmic Nrf2 levels compared to H_2_O_2_-treated group (*P* < 0.05). Phosphatidylinositol-3-kinase (PI3K)/Akt pathway is an important upstream regulator of Nrf2 signaling that promotes Nrf2 phosphorylation and nuclear translocation [25–27]. We found that Akt phosphorylation in H_2_O_2_-stimulated cells was overtly lower (*P* < 0.01 vs. control group). Mep-S or NAC pretreatment significantly suppressed this effect compared with H_2_O_2_-challenged group (*P* < 0.05).

It has been suggested that small-molecule regulators can enhance Nrf2 signaling through directly binding with Keap1 or Nrf2 to impair Keap1-Nrf2 PPI [28, 29]. We next performed molecular docking analysis to investigate whether Mep-S could affect PPI between Keap1 and Nrf2. As demonstrated in Figure 4(b), Mep-S inserted into the central pocket containing the Nrf2 binding domain and established multiple interactions with several key residues of Kelch domain in mouse Keap1. Its benzene ring established a *π*–*π* stacking interaction with Tyr334. The phenolic hydroxyl established a favorable hydrogen bond with Ser602. Additionally, the nitrogen in the indole ring established two hydrogen bonds with Val418 and Val465. Meanwhile, the hydroxyl group at the 5 position interacted with Val606 and Gly367 via three hydrogen bonds. These results indicate that Mep-S may bind to the Kelch domain of Keap1 to disrupt the Keap1-Nrf2 PPI.

### 3.5. Mep-S Attenuates Scopolamine-Induced Cognitive Deficits in Mice

**Figure 5:**
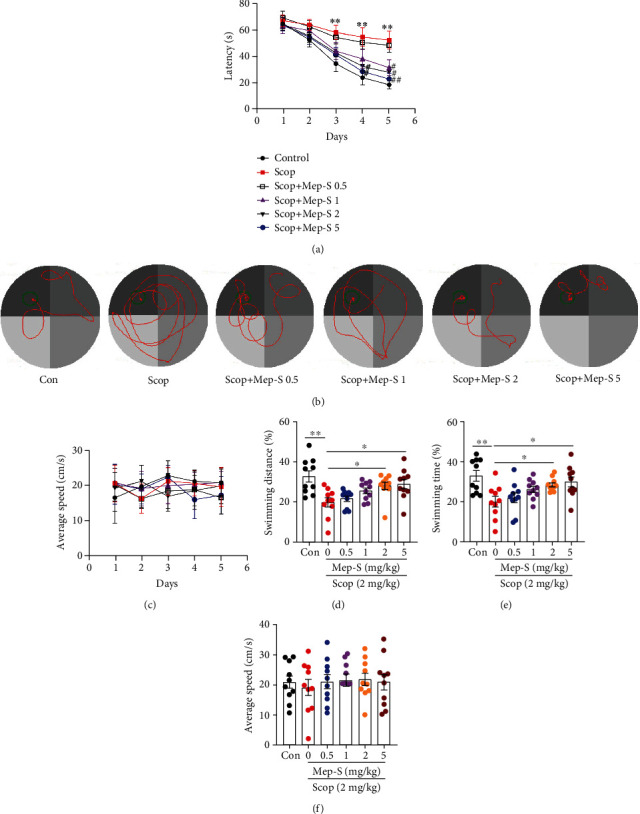
Mep-S improves scopolamine-induced learning and memory dysfunction in mice. The escape latency to the platform (a), swimming tracks (b), and average swimming speeds (c) of mice during the 5-day training sessions. ^∗∗^*P* < 0.01 vs. control group; ^#^*P* < 0.05 and ^##^*P* < 0.01 vs. scopolamine-treated group. The proportions of swimming distance (d) and time (e) in the target quadrant and average swimming speeds (f) of mice during the probe trial on day 6. One-way ANOVA: *F*(5, 54) = 5.30, *P* = 0.0005 (distance); *F*(5, 54) = 4.89, *P* = 0.009 (time); *F*(5, 54) = 0.17, *P* = 0.97 (speeds). Data are expressed as the mean ± SEM (*n* = 10). ^∗^*P* < 0.05 and ^∗∗^*P* < 0.01.

Based on the *in vitro* neuroprotective effect of Mep-S, we subsequently examined whether Mep-S could enhance learning ability in scopolamine-challenged mice. The Morris water maze task was conducted to assess whether Mep-S could improve the hippocampus-dependent learning capacity. Two-way ANOVA showed overt effects of Mep-S treatment (*F*(5, 270) = 12.50) and training days (*F*(4, 270) = 39.61) on escape latency (both *P* < 0.0001). As demonstrated in Figures 5(a) and 5(b), control mice displayed gradually reduced escape latency from 64.40 s to 18.48 s over the 5 consecutive training days. Mice in the scopolamine-treated group showed longer latency (from 66.77 s to 52.10 s) over the training days (*P* < 0.01 vs. control group on days 3-5), suggesting that scopolamine induced an impairment of spatial memory. The latency on days 4 and 5 in mice pretreated with Mep-S (1, 2, or 5 mg/kg) was obviously shorter compared with scopolamine-challenged animals. The average swimming speeds of mice during the 5-day training sessions were similar among groups (two-way ANOVA, *F*(5, 270) = 0.15, *P* = 0.98 for Mep-S treatment and *F*(4, 270) = 0.10, *P* = 0.98 for days; Figure 5(c)). As shown in Figures 5(d) and 5(e), during the probe test on the 6th day, scopolamine-treated mice displayed markedly decreased proportions of swimming distance and time in target quadrant (*P* < 0.01 vs. control animals). In mice receiving Mep-S (2 or 5 mg/kg) pretreatment, the proportions of swimming distance and time in target quadrant were significantly higher (*P* < 0.05 vs. scopolamine-challenged mice). The average swimming speeds of mice on day 6 showed no significant difference between groups (Figure 5(f)). Mice did not show thigmotaxis during the Morris water maze test (data not shown).

### 3.6. Mep-S Attenuates Neuronal Apoptosis and Inhibits AChE Activity in Scopolamine-Challenged Mice

**Figure 6:**
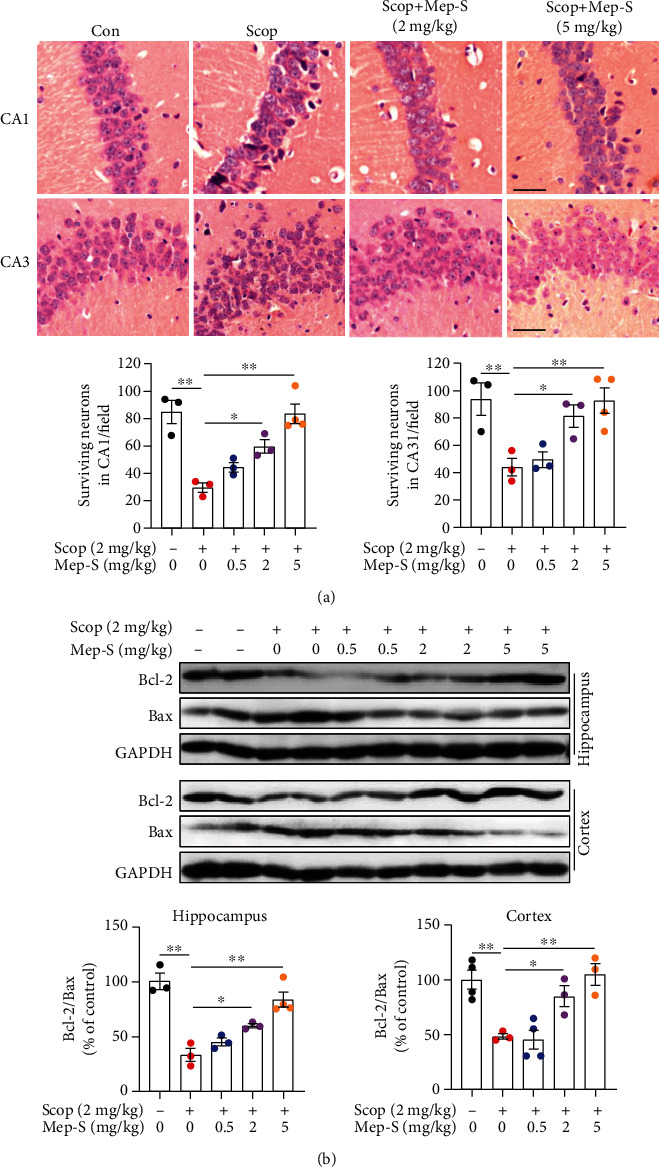
Mep-S attenuates neuronal apoptosis in the brains of scopolamine-treated mice. (a) Representative images of hematoxylin and eosin staining (upper panel) and quantitative analysis of the number of surviving neurons (lower panel) in the hippocampal CA1 and CA3 regions. Scale bar, 50 *μ*m. One-way ANOVA: *F*(4, 11) = 16.15, *P* = 0.0001 (CA1); *F*(4, 11) = 7.17, *P* = 0.0043 (CA3). (b) Protein expression of Bcl-2 and Bax in the hippocampus and cerebral cortex of mice was determined by western blot analysis. One-way ANOVA: *F*(4, 11) = 21.66, *P* < 0.0001 (hippocampus); *F*(4, 12) = 11.08, *P* = 0.0005 (cortex). Data are presented as the mean ± SEM (*n* = 3 − 4 per group). ^∗^*P* < 0.05 and ^∗∗^*P* < 0.01.

We subsequently detected whether Mep-S could attenuate scopolamine-induced neuronal death in brain. H&E staining revealed markedly lower amount of surviving neuronal cells in CA1 and CA3 regions of hippocampus in mice receiving scopolamine challenge (*P* < 0.01 vs. control mice). Pretreatment with Mep-S at 2 and 5 mg/kg markedly inhibited the scopolamine-induced decrease in surviving neuronal number in mouse hippocampus (Figure 6(a)). We then examined the expression of some antiapoptotic and apoptotic markers. It was found that scopolamine reduced expression of the antiapoptotic protein Bcl-2 and enhanced expression of the apoptotic protein Bax, leading to decreased Bcl-2 to Bax ratios in mouse hippocampus and cortex (*P* < 0.01 vs. the control animals). Mep-S pretreatment resulted in enhanced Bcl-2 levels and reduced Bax levels, leading to increased Bcl-2 to Bax ratios in the hippocampus (2 mg/kg: by 0.81-fold, *P* < 0.05; 5 mg/kg: by 1.53-fold, *P* < 0.01) and cortex (2 mg/kg: by 0.76-fold, *P* < 0.05; 5 mg/kg: by 1.16-fold, *P* < 0.01) of mice relative to those in mice receiving scopolamine alone (Figure 6(b)). These results indicate that Mep-S attenuated scopolamine-induced neuronal apoptosis *in vivo*.

We also measured AChE activity in mouse brains. Brain AChE activity showed obviously between-group difference (one-way ANOVA, *F*(4, 15) = 13.00, *P* < 0.0001; Supplementary material, Figure S2). AChE activity in brain tissues markedly elevated in mice challenged with scopolamine (*P* < 0.01 vs. control group). Brain AChE activity in mice pretreated with Mep-S was markedly lower compared to that of mice receiving scopolamine alone (*P* < 0.05).

### 3.7. Antioxidant Effect of Mep-S via Activating the Nrf2/Antioxidant Enzyme Pathway in Scopolamine-Challenged Mice

**Figure 7:**
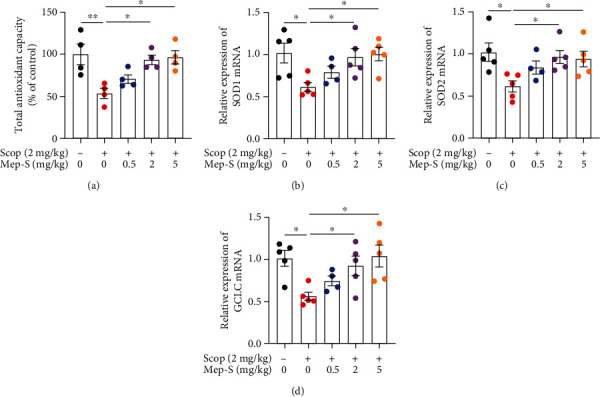
Effect of Mep-S on oxidative status in the brains of scopolamine-treated mice. (a) The total antioxidant capacity in brain tissues of mice. One-way ANOVA: *F*(4, 15) = 6.49, *P* = 0.0031.The mRNA levels of SOD1 (b), SOD2 (c), and GCLC (d) in the brain tissues of mice. One-way ANOVA: *F*(4, 19) = 3.76, *P* = 0.020 (SOD1); *F*(4, 19) = 3.46, *P* = 0.028 (SOD2); *F*(4, 19) = 4.38, *P* = 0.011 (GCLC). Data are presented as the mean ± SEM (*n* = 4 − 5 per group). ^∗^*P* < 0.05 and ^∗∗^*P* < 0.01.

**Figure 8:**
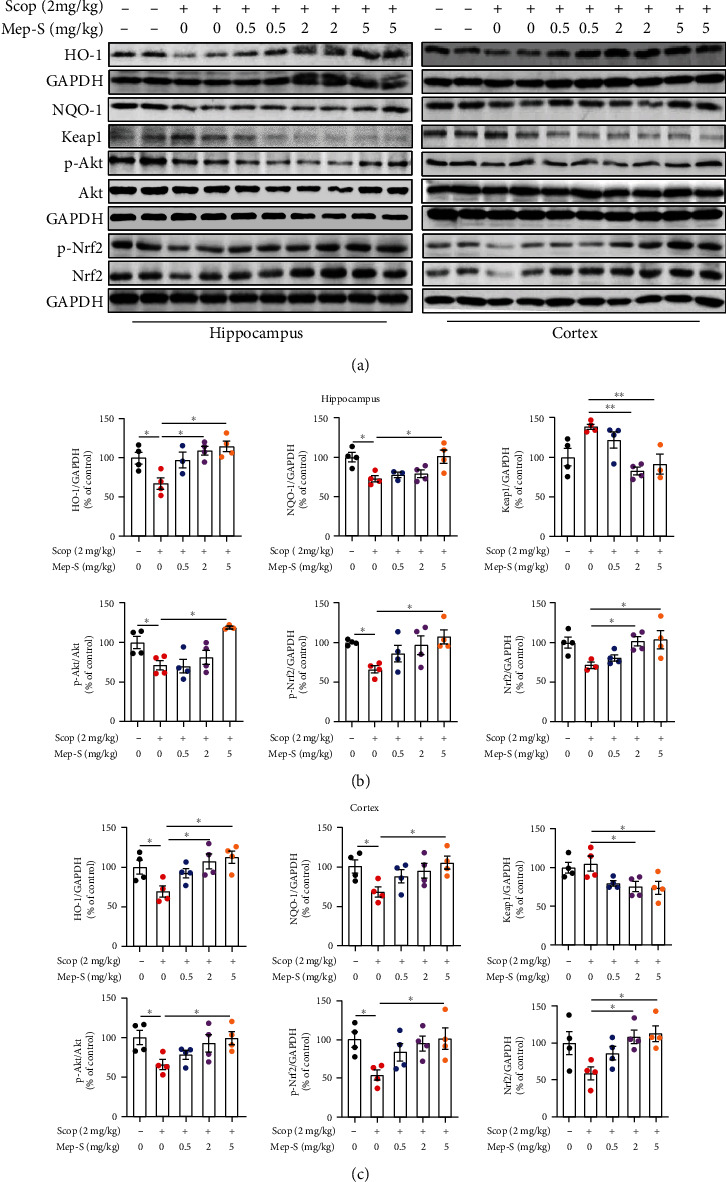
Mep-S activates the Nrf2/antioxidant enzyme pathway in the brains of scopolamine-treated mice. (a) Protein levels of HO-1, NQO-1, Keap1, and phosphorylated Akt and Nrf2 in the hippocampus and cerebral cortex of mice detected by western blot analysis. Densitometric analysis of protein expression in the Nrf2/antioxidant enzyme pathway in the hippocampus (b) and cortex (c) of mice in the control, scopolamine-treated, and scopolamine+Mep-S-treated groups. One-way ANOVA for hippocampus: *F*(4, 14) = 6.58, *P* = 0.0034 (HO-1); *F*(4, 14) = 5.51, *P* = 0.071 (NQO-1); *F*(4, 14) = 6.97, *P* = 0.0026 (Keap1); *F*(4, 14) = 7.16, *P* = 0.0023 (p-Akt); *F*(4, 15) = 3.52, *P* = 0.0032 (p-Nrf2); *F*(4, 14) = 3.67, *P* = 0.030 (Nrf2). One-way ANOVA for cortex: *F*(4, 15) = 4.59, *P* = 0.013(HO-1); *F*(4, 15) = 3.10, *P* = 0.048 (NQO-1); *F*(4, 15) = 4.08, *P* = 0.020 (Keap1); *F*(4, 15) = 3.18, *P* = 0.044 (p-Akt); *F*(4, 15) = 3.38, *P* = 0.037 (p-Nrf2); *F*(4, 15) = 3.87, *P* = 0.024 (Nrf2). Data are presented as the mean ± SEM (*n* = 3 − 4 per group). ^∗^*P* < 0.05 and ^∗∗^*P* < 0.01.

We next detected the effect of Mep-S on brain oxidative status in scopolamine-treated mice. As shown in Figure 7, the total antioxidant capacity along with mRNA levels of SOD1, SOD2, and GCLC in brain tissues markedly declined in scopolamine-treated mice compared with the control mice. Mep-S markedly inhibited the scopolamine-induced effects (*P* < 0.05). Moreover, levels of HO-1, NQO-1, and phosphorylated Akt and Nrf2 in the hippocampal and cortex regions were lower in scopolamine-challenged mice than in control mice (*P* < 0.05). Mep-S at 5 mg/kg resulted in elevated levels of these proteins in mouse hippocampal and cortex regions (*P* < 0.05 vs. mice receiving scopolamine alone). Mep-S at 2 mg/kg led to elevated levels of HO-1 and Nrf2 in the hippocampus and cortex (*P* < 0.05 vs. mice receiving scopolamine alone). Scopolamine did not obviously affect Keap1 and Nrf2 levels. However, mice receiving Mep-S (2 and 5 mg/kg) treatment showed decreased Keap1 levels and increased Nrf2 expression in mouse hippocampal and cortex regions relative to mice receiving scopolamine alone (Figure 8). The results of immunofluorescence staining showed that scopolamine decreased Nrf2 immunoreactivity in mouse hippocampal region, while Mep-S (5 mg/kg) overtly counteracted this effect (Supplementary material, Figure S3).

## 4. Discussion

AD is a neurodegenerative disease associated with multiple etiological factors. As oxidative stress is a key mechanism for AD-associated neuronal injury [7], pharmacological interventions targeting oxidative neurotoxicity may be an important strategy for AD treatment. Here, we demonstrate that Mep-S, a novel multifunctional (-)-meptazinol-serotonin hybrid with the potential for Keap1-Nrf2 PPI inhibition, attenuates apoptotic neuronal death and memory dysfunction associated with oxidative stress. These effects may be exerted through activating Nrf2/antioxidant enzyme axis. Therefore, Mep-S may be a potential MTDL lead with antioxidant activity for the intervention of AD-like symptoms.

The brain, with high oxygen consumption, is especially at risk of oxidative stress [30]. ROS (including superoxide, hydroxyl radicals, H_2_O_2_, and singlet oxygen) are regarded as key factors for oxidative pathology [26, 31, 32]. In *in vitro* experiments, we examined the effect of Mep-S in H_2_O_2_-stimulated neuronal cells. The results showed that Mep-S inhibited H_2_O_2_-induced cytotoxicity in SH-SY5Y cells, as demonstrated with increased cell viability and decreased LDH release. Furthermore, Mep-S suppressed H_2_O_2_-induced neuronal apoptosis (detected by Annexin V/PI staining) by restoring MMP and inhibiting caspase-3 cleavage. These findings indicate a neuroprotective effect of Mep-S *in vitro*. We subsequently detected whether Mep-S attenuates oxidative stress trigger by H_2_O_2_. We found that Mep-S suppressed H_2_O_2_-induced intracellular ROS accumulation (detected by the fluorescence probe DCFH-DA) in neuronal cells. Moreover, the reduction in GSH content and SOD activity caused by H_2_O_2_ was markedly inhibited by Mep-S. These results suggest that Mep-S repressed H_2_O_2_-elicited oxidative neuronal damage, and the antioxidant effect may contribute to the neuroprotective effect of Mep-S.

The Nrf2/antioxidant enzyme pathway is essential for cells to prevent oxidative injury [27, 33]. Physiologically, intracellular Nrf2 level is controlled by the cytosolic repressor Keap1, which can promote ubiquitination and the following proteasomal degradation of Nrf2 via PPI between Keap1 and Nrf2. During the oxidative stress-triggered adaptive responses, Keap1 is inactivated due to a conformational change, facilitating Nrf2 dissociation from the Keap1-Nrf2 complex and nuclear translocation to bind to the antioxidant response element (ARE) and transcriptionally induce expression of antioxidant enzymes [34, 35]. However, overproduction of ROS may cause deregulation of redox balance and, thus, hinder nuclear translocation of Nrf2, resulting in impaired antioxidant defense responses [15, 36, 37]. Accordingly, enhancement of Keap1/Nrf2 signaling is reported to protect against AD-associated neurodegeneration and memory dysfunction by restoring redox homeostasis, and thus, this pathway may be an attractive drug target for AD treatment [24, 38, 39]. In addition, activation of this pathway contributes to the neuroprotective effect of bioactive agents in animal models of other neurological disorders, such as Parkinson's disease and traumatic brain injury [40, 41]. To get a deeper understanding of the mechanisms for neuroprotection of Mep-S, we explored whether Mep-S influences this pathway. We found that in H_2_O_2_-challenged SH-SY5Y cells, Mep-S promoted expression of HO-1 and NQO-1. Meanwhile, Mep-S reduced the protein level of Keap1 while promoted nuclear translocation of Nrf2. Therefore, Mep-S could attenuate oxidative neurotoxicity via enhancing Keap1/Nrf2/antioxidant enzyme signaling. It was also observed that Mep-S promoted Akt phosphorylation in H_2_O_2_-challenged SH-SY5Y cells. Since PI3K/Akt signaling can facilitate Nrf2 phosphorylation, thereby promoting its dissociation from Keap1 [25–27, 35], our results indicate that Akt activation may also be involved in Nrf2 activation induced by Mep-S. The *in vitro* and *in vivo* studies have reported that Keap1-Nrf2 PPI inhibitors protect neuronal functioning in AD [38, 39]. We next performed molecular docking simulations to explore whether Mep-S could directly disrupt the Keap1-Nrf2 PPI. It was shown that Mep-S can form a *π*–*π* stacking interaction with Tyr334 and form hydrogen bonds with Ser602, Val418, Val465, Val606, and Gly367 of the Keap1 Kelch domain at the Keap1-Nrf2 interface. These results indicate that Mep-S directly binds with Keap1 to interfere with Keap1-Nrf2 PPI, leading to Nrf2 activation. Taken together, the above results suggest that Mep-S ameliorates oxidative neuronal damage via enhancing Nrf2/antioxidant enzyme signaling, in which Mep-S may promote the separation of Nrf2 from Keap1 (by inactivating Keap1) and phosphorylation of Nrf2 (by activating PI3K/Akt axis).

Scopolamine can easily enter into the brain to block muscarinic acetylcholine receptors. It has been applied to elicit memory dysfunction in AD-associated animal experiments. It induces cholinergic dysfunction and increases A*β* and tau deposition in the central nervous system [42–44]. It also causes mitochondrial dysfunction and neuroinflammation [44]. Moreover, it causes oxidative stress and promotes neuronal apoptosis [15, 44, 45]. Therefore, scopolamine has been applied in animal models to elicit AD-like symptoms involving both cholinergic dysfunction and oxidative stress [15, 18]. We first performed behavioral experiments using the Morris water maze test to detect the effect of Mep-S in scopolamine-challenged mice. It was found that scopolamine-treated mice displayed cognitive dysfunction with impaired acquisition and retention of spatial memory, while Mep-S protected against scopolamine-induced cognitive deficits. Further experiments revealed that Mep-S improved the survival of hippocampal neurons and increased Bcl-2 to Bax ratios (an antiapoptotic index) in the hippocampal and cortex regions of scopolamine-challenged mice, indicating that the effect of Mep-S on scopolamine-induced AD-like symptoms may be associated with its protection against neuronal apoptosis.

We next examined whether the antioxidant activity of Mep-S could have a role in its *in vivo* effect. The total antioxidant capacity as well as expression of SOD1, SOD2, and GCLC (the rate-limiting enzyme for GSH synthesis [46]) in mouse brains decreased after scopolamine exposure, while Mep-S suppressed the effect of scopolamine. These results indicate that Mep-S could provide protection against scopolamine-induced oxidative neurotoxicity. To investigate the underlying mechanisms, the effect of Mep-S on the Nrf2/antioxidant enzyme pathway was tested. As expected, Mep-S increased HO-1 and NQO-1 expression along with the levels of Nrf2 and phosphorylated Akt and Nrf2, accompanied with reduced Keap1 levels in the hippocampal and cortex regions of scopolamine-challenged mice. Collectively, the above findings indicate that the nootropic effect of Mep-S in the scopolamine-elicited cognitive dysfunction model may involve neuroprotection against oxidative stress. We also observed that Mep-S suppressed scopolamine-promoted AChE activity in mouse brains. Notably, evidence suggests an association between brain AChE activity and oxidative stress [17, 47]. Therefore, the inhibition of AChE activity by Mep-S could also contribute to neuroprotective and nootropic effects by attenuating oxidative neurotoxicity in the brains of scopolamine-treated mice, in addition to improving neurotransmission by elevating acetylcholine levels in the synaptic cleft.

Our research has some limitations. We only used male mice to explore the protection of Mep-S. Notably, both sexes are appropriate in AD research, and female animals may be more prominent since AD shows a higher incidence in women [48, 49]. Evidence shows that male and female animals exhibit similar variability of proxies for physiological and neurological outputs across multiple timescales [50]. In contrast, recent studies demonstrate that cognitive impairment of AD animals were detected in males but not in females [51, 52]. Therefore, sex differences should also be considered in the exploration of neuroprotective mechanisms. Since our results cannot reflect drug effect in females, future research is needed to investigate the potential of Mep-S for AD therapy in female AD models. In addition, although scopolamine have been used to induce memory dysfunction in animal models [15, 18], the pathological changes and cognitive impairment caused by scopolamine may not precisely mimic AD pathology and thus are not specific for AD. In other words, it only induces AD-like pathology and symptoms. Researchers are using transgenic AD animal models to evaluate the therapeutic effect of MTDLs [53]. Therefore, future studies using transgenic AD animal models are warranted.

**Figure 9:**
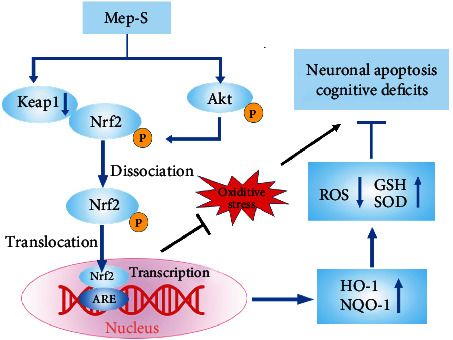
Proposed schematic summary of the protective effect of Mep-S against oxidative stress-mediated neuronal apoptosis and cognitive deficits. Mep-S promotes the nuclear translocation of Nrf2 to transcriptionally induce the expression of antioxidant enzymes such as HO-1 and NQO-1 by promoting the dissociation of Nrf2 from Keap1 by inactivating Keap1 and the phosphorylation of Nrf2 by activating Akt. The activation of the Nrf2/antioxidant enzyme pathway causes decreased ROS levels, increased GSH levels and SOD activity, and enhanced oxidant resistance, which may contribute to the protective effect of Mep-s against oxidative stress-mediated neuronal apoptosis and cognitive deficits.

Collectively, this work demonstrates that Mep-S, a potential Keap1-Nrf2 PPI blocker, attenuates apoptotic neuronal death and scopolamine-elicited memory dysfunction associated with oxidative stress. These effects could be exerted via enhancement of Nrf2/antioxidant enzyme signaling by promoting separation of Nrf2 from Keap1 by inactivating Keap1 and phosphorylation of Nrf2 by activating Akt (Figure 9). Additionally, it inhibits brain AChE activity to improve neurotransmission impairment and oxidative neuronal injury in scopolamine-treated mice. Therefore, Mep-S may be a potential lead for multitarget neuroprotective agents to treat AD-like symptoms.

## Data Availability

Data in support of the observations of this work were included in the article and the supplementary files.

## Supplementary Materials

Supplementary Materials Supplementary Figure 1: The viability of SH-SY5Y cells treated with the indicated concentrations of Mep-S (A) or H_2_O_2_ (B) for 24 h. Data are presented as the mean ± SEM of 3-4 independent experiments. ^∗^*P* < 0.05 and ^∗∗^*P* < 0.01 compared with the control group. Supplementary Figure 2: Measurement of acetylcholinesterase (AChE) activity in mouse brains. After 6-day scopolamine treatment, AChE activity was determined in homogenates of brain tissues of mice in each group. Data are presented as the mean ± SEM (*n* = 3 − 5 per group). ^∗^*P* < 0.05 and ^∗∗^*P* < 0.01. Supplementary Figure 3: Representative photographs of immunofluorescence staining of Nrf2 (green) and DAPI (blue) in mouse hippocampus of the control, scopolamine-treated, and scopolamine+Mep-S-treated groups.

## References

[1] Long J. M., Holtzman D. M. (2019). Alzheimer disease: an update on pathobiology and treatment strategies. *Cell*.

[2] Ferris S. H., Farlow M. (2013). Language impairment in Alzheimer’s disease and benefits of acetylcholinesterase inhibitors. *Clinical Interventions in Aging*.

[3] McHardy S. F., Wang H. L., McCowen S. V., Valdez M. C. (2017). Recent advances in acetylcholinesterase inhibitors and reactivators: an update on the patent literature (2012-2015). *Expert Opinion on Therapeutic Patents*.

[4] Malinow R. (2012). New developments on the role of NMDA receptors in Alzheimer's disease. *Current Opinion in Neurobiology*.

[5] Graham W. V., Bonito-Oliva A., Sakmar T. P. (2017). Update on Alzheimer’s disease therapy and prevention strategies. *Annual Review of Medicine*.

[6] Rabinovici G. D. (2021). Controversy and progress in Alzheimer's disease - FDA approval of aducanumab. *The New England Journal of Medicine*.

[7] Butterfield D. A., Halliwell B. (2019). Oxidative stress, dysfunctional glucose metabolism and Alzheimer disease. *Nature Reviews. Neuroscience*.

[8] Jiang T., Sun Q., Chen S. (2016). Oxidative stress: a major pathogenesis and potential therapeutic target of antioxidative agents in Parkinson's disease and Alzheimer's disease. *Progress in Neurobiology*.

[9] Kamat P. K., Kalani A., Rai S. (2016). Mechanism of oxidative stress and synapse dysfunction in the pathogenesis of Alzheimer's disease: understanding the therapeutics strategies. *Molecular Neurobiology*.

[10] Cheignon C., Tomas M., Bonnefont-Rousselot D., Faller P., Hureau C., Collin F. (2018). Oxidative stress and the amyloid beta peptide in Alzheimer's disease. *Redox Biology*.

[11] Mecocci P., Polidori M. C. (2012). Antioxidant clinical trials in mild cognitive impairment and Alzheimer's disease. *Biochimica et Biophysica Acta*.

[12] Wojsiat J., Zoltowska K. M., Laskowska-Kaszub K., Wojda U. (2018). Oxidant/antioxidant imbalance in Alzheimer’s disease: therapeutic and diagnostic prospects. *Oxidative Medicine and Cellular Longevity*.

[13] Veurink G., Perry G., Singh S. K. (2020). Role of antioxidants and a nutrient rich diet in Alzheimer's disease. *Open Biology*.

[14] Wu W. Y., Dai Y. C., Li N. G. (2017). Novel multitarget-directed tacrine derivatives as potential candidates for the treatment of Alzheimer's disease. *Journal of Enzyme Inhibition and Medicinal Chemistry*.

[15] Wan T., Wang Z., Luo Y. (2019). FA-97, a new synthetic caffeic acid phenethyl ester derivative, protects against oxidative stress-mediated neuronal cell apoptosis and scopolamine- induced cognitive impairment by activating Nrf2/HO-1 signaling. *Oxidative Medicine and Cellular Longevity*.

[16] Sang Z., Wang K., Shi J. (2020). Apigenin-rivastigmine hybrids as multi-target-directed liagnds for the treatment of Alzheimer's disease. *European Journal of Medicinal Chemistry*.

[17] Walczak-Nowicka Ł. J., Herbet M. (2021). Acetylcholinesterase inhibitors in the treatment of neurodegenerative diseases and the role of acetylcholinesterase in their pathogenesis. *International Journal of Molecular Sciences*.

[18] Park J. W., Kim J. E., Kang M. J. (2019). Anti-oxidant activity of gallotannin-enriched extract of Galla Rhois can associate with the protection of the cognitive impairment through the regulation of BDNF signaling pathway and neuronal cell function in the scopolamine-treated ICR mice. *Antioxidants (Basel).*.

[19] Cheng S., Zheng W., Gong P. (2015). (−)-Meptazinol-melatonin hybrids as novel dual inhibitors of cholinesterases and amyloid-*β* aggregation with high antioxidant potency for Alzheimer's therapy. *Bioorganic & Medicinal Chemistry*.

[20] Pallesen J. S., Narayanan D., Tran K. T. (2021). Deconstructing noncovalent Kelch-like ECH-associated protein 1 (Keap1) inhibitors into fragments to reconstruct new potent compounds. *Journal of Medicinal Chemistry*.

[21] Zhong M., Lynch A., Muellers S. N. (2020). Interaction energetics and druggability of the protein-protein interaction between Kelch-like ECH-associated protein 1 (KEAP1) and nuclear factor erythroid 2 like 2 (Nrf2). *Biochemistry*.

[22] Guan H., Li J., Tan X. (2020). Natural xanthone *α*-mangostin inhibits LPS-induced microglial inflammatory responses and memory impairment by blocking the TAK1/NF-*κ*B signaling pathway. *Molecular Nutrition & Food Research*.

[23] Bock F. J., Tait S. W. G. (2020). Mitochondria as multifaceted regulators of cell death. *Nature Reviews. Molecular Cell Biology*.

[24] Fão L., Mota S. I., Rego A. C. (2019). Shaping the Nrf2-ARE-related pathways in Alzheimer's and Parkinson's diseases. *Ageing Research Reviews*.

[25] Yoo J. M., Lee B. D., Sok D. E., Ma J. Y., Kim M. R. (2017). Neuroprotective action of N-acetyl serotonin in oxidative stress-induced apoptosis through the activation of both TrkB/CREB/BDNF pathway and Akt/Nrf2/antioxidant enzyme in neuronal cells. *Redox Biology*.

[26] Li S. T., Dai Q., Zhang S. X. (2018). Ulinastatin attenuates LPS-induced inflammation in mouse macrophage RAW264.7 cells by inhibiting the JNK/NF-*κ*B signaling pathway and activating the PI3K/Akt/Nrf2 pathway. *Acta Pharmacologica Sinica*.

[27] de Freitas S. M., Pruccoli L., Morroni F. (2018). The keap1/Nrf2-ARE pathway as a pharmacological target for chalcones. *Molecules*.

[28] Abed D. A., Goldstein M., Albanyan H., Jin H., Hu L. (2015). Discovery of direct inhibitors of Keap1-Nrf2 protein-protein interaction as potential therapeutic and preventive agents. *Acta Pharmaceutica Sinica B*.

[29] Zhuang C., Wu Z., Xing C., Miao Z. (2017). Small molecules inhibiting Keap1-Nrf2 protein-protein interactions: a novel approach to activate Nrf2 function. *MedChemComm*.

[30] Luca M., Di Mauro M., Di Mauro M., Luca A. (2019). Gut microbiota in Alzheimer’s disease, depression, and type 2 diabetes mellitus: the role of oxidative stress. *Oxidative Medicine and Cellular Longevity*.

[31] Dikalov S. I., Harrison D. G. (2014). Methods for detection of mitochondrial and cellular reactive oxygen species. *Antioxidants & Redox Signaling*.

[32] Sies H. (2017). Hydrogen peroxide as a central redox signaling molecule in physiological oxidative stress: oxidative eustress. *Redox Biology*.

[33] Zhang Z., Zhou S., Jiang X. (2015). The role of the Nrf2/Keap1 pathway in obesity and metabolic syndrome. *Reviews in Endocrine & Metabolic Disorders*.

[34] Tonelli C., Chio I. I. C., Tuveson D. A. (2018). Transcriptional regulation by Nrf2. *Antioxidants & Redox Signaling*.

[35] Yu C., Xiao J. H. (2021). The Keap1-Nrf2 system: a mediator between oxidative stress and aging. *Oxidative Medicine and Cellular Longevity*.

[36] Sies H., Jones D. P. (2020). Reactive oxygen species (ROS) as pleiotropic physiological signalling agents. *Nature Reviews. Molecular Cell Biology*.

[37] Guo S., Zhang Q. (2021). Paeonol protects melanocytes against hydrogen peroxide-induced oxidative stress through activation of Nrf2 signaling pathway. *Drug Development Research*.

[38] Dumont M., Wille E., Calingasan N. Y. (2009). Triterpenoid CDDO-methylamide improves memory and decreases amyloid plaques in a transgenic mouse model of Alzheimer’s disease. *Journal of Neurochemistry*.

[39] Kerr F., Sofola-Adesakin O., Ivanov D. K. (2017). Direct Keap1-Nrf2 disruption as a potential therapeutic target for Alzheimer's disease. *PLoS Genetics*.

[40] Wang Y., Gao L., Chen J. (2021). Pharmacological modulation of Nrf2/HO-1 signaling pathway as a therapeutic target of Parkinson's disease. *Frontiers in Pharmacology*.

[41] Salman M., Tabassum H., Parvez S. (2020). Nrf2/HO-1 mediates the neuroprotective effects of pramipexole by attenuating oxidative damage and mitochondrial perturbation after traumatic brain injury in rats. *Disease Models & Mechanisms*.

[42] Pinto T., Lanctôt K. L., Herrmann N. (2011). Revisiting the cholinergic hypothesis of behavioral and psychological symptoms in dementia of the Alzheimer's type. *Ageing Research Reviews*.

[43] Chen W. N., Yeong K. Y. (2020). Scopolamine, a toxin-induced experimental model, used for research in Alzheimer's disease. *CNS & Neurological Disorders Drug Targets*.

[44] Tang K. S. (2019). The cellular and molecular processes associated with scopolamine-induced memory deficit: a model of Alzheimer's biomarkers. *Life Sciences*.

[45] Muhammad T., Ali T., Ikram M., Khan A., Alam S. I., Kim M. O. (2019). Melatonin rescue oxidative stress-mediated neuroinflammation/neurodegeneration and memory impairment in scopolamine-induced amnesia mice model. *Journal of Neuroimmune Pharmacology*.

[46] Yang Y., Dieter M. Z., Chen Y., Shertzer H. G., Nebert D. W., Dalton T. P. (2002). Initial characterization of the glutamate-cysteine ligase modifier subunit *Gclm*−/− knockout mouse:. *The Journal of Biological Chemistry*.

[47] Wang X., Li P., Ding Q., Wu C., Zhang W., Tang B. (2019). Observation of acetylcholinesterase in stress-induced depression phenotypes by two-photon fluorescence imaging in the mouse brain. *Journal of the American Chemical Society*.

[48] Fisher D. W., Bennett D. A., Dong H. (2018). Sexual dimorphism in predisposition to Alzheimer's disease. *Neurobiology of Aging*.

[49] Salman M., Akram M., Shahrukh M., Ishrat T., Parvez S. (2022). Effects of pramipexole on beta-amyloid_1-42_ memory deficits and evaluation of oxidative stress and mitochondrial function markers in the hippocampus of Wistar rat. *Neurotoxicology*.

[50] Smarr B. L., Grant A. D., Zucker I., Prendergast B. J., Kriegsfeld L. J. (2017). Sex differences in variability across timescales in BALB/c mice. *Biology of Sex Differences*.

[51] Bao J., Mahaman Y. A. R., Liu R. (2017). Sex differences in the cognitive and hippocampal effects of streptozotocin in an animal model of sporadic AD. *Frontiers in Aging Neuroscience*.

[52] Pilipenko V., Narbute K., Amara I. (2019). GABA-containing compound gammapyrone protects against brain impairments in Alzheimer’s disease model male rats and prevents mitochondrial dysfunction in cell culture. *Journal of Neuroscience Research*.

[53] Qiu H., Liu X. (2022). Echinacoside improves cognitive impairment by inhibiting A*β* deposition through the PI3K/AKT/Nrf2/PPAR*γ* signaling pathways in APP/PS1 mice. *Molecular Neurobiology*.

