# Supplementary material for: A Multifunctional (-)-Meptazinol-Serotonin Hybrid Ameliorates Oxidative Stress-Associated Apoptotic Neuronal Death and Memory Deficits via Activating the Nrf2/Antioxidant Enzyme Pathway
Source: Oxid Med Cell Longev. 2023 Feb 9;2023:6935947. doi: 10.1155/2023/6935947 (PMC9935814; doi:10.1155/2023/6935947)
Supplement: Supplementary Materials — Supplementary Figure 1: The viability of SH-SY5Y cells treated with the indicated concentrations of Mep-S (A) or H2O2 (B) for 24 h. Data are presented as the mean ± SEM of 3-4 independent experiments. ∗P < 0.05 and ∗∗P < 0.01 compared with the control group. Supplementary Figure 2: Measurement of acetylcholinesterase (AChE) activity in mouse brains. After 6-day scopolamine treatment, AChE activity was determined in homogenates of brain tissues of mice in each group. Data are presented as the mean ± SEM (n = 3 − 5 per group). ∗P < 0.05 and ∗∗P < 0.01. Supplementary Figure 3: Representative photographs of immunofluorescence staining of Nrf2 (green) and DAPI (blue) in mouse hippocampus of the control, scopolamine-treated, and scopolamine+Mep-S-treated groups. [file 6935947.f1.docx]

**A**

**B**

Figure S1. The viability of SH-SY5Y cells treated with the indicated concentrations of Mep-S (A) or H_2_O_2_ (B) for 24 h. Data are presented as the mean ± SEM of 3-4 independent experiments. * *P*< 0.05, ** *P*< 0.01 compared with the control group.


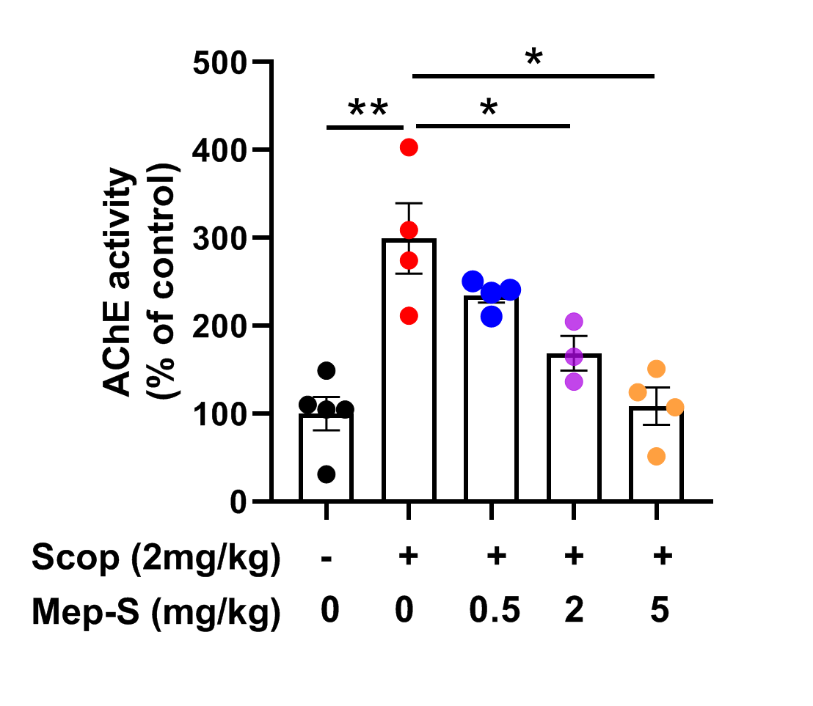


FigureS2. Measurement of acetylcholinesterase (AChE) activity in mouse brains. After 6-day scopolamine treatment, AChE activity was determined in homogenates of brain tissues of mice in each group. Data are presented as the mean ± SEM (n = 3-5 per group). **P*< 0.05, ***P*< 0.01.


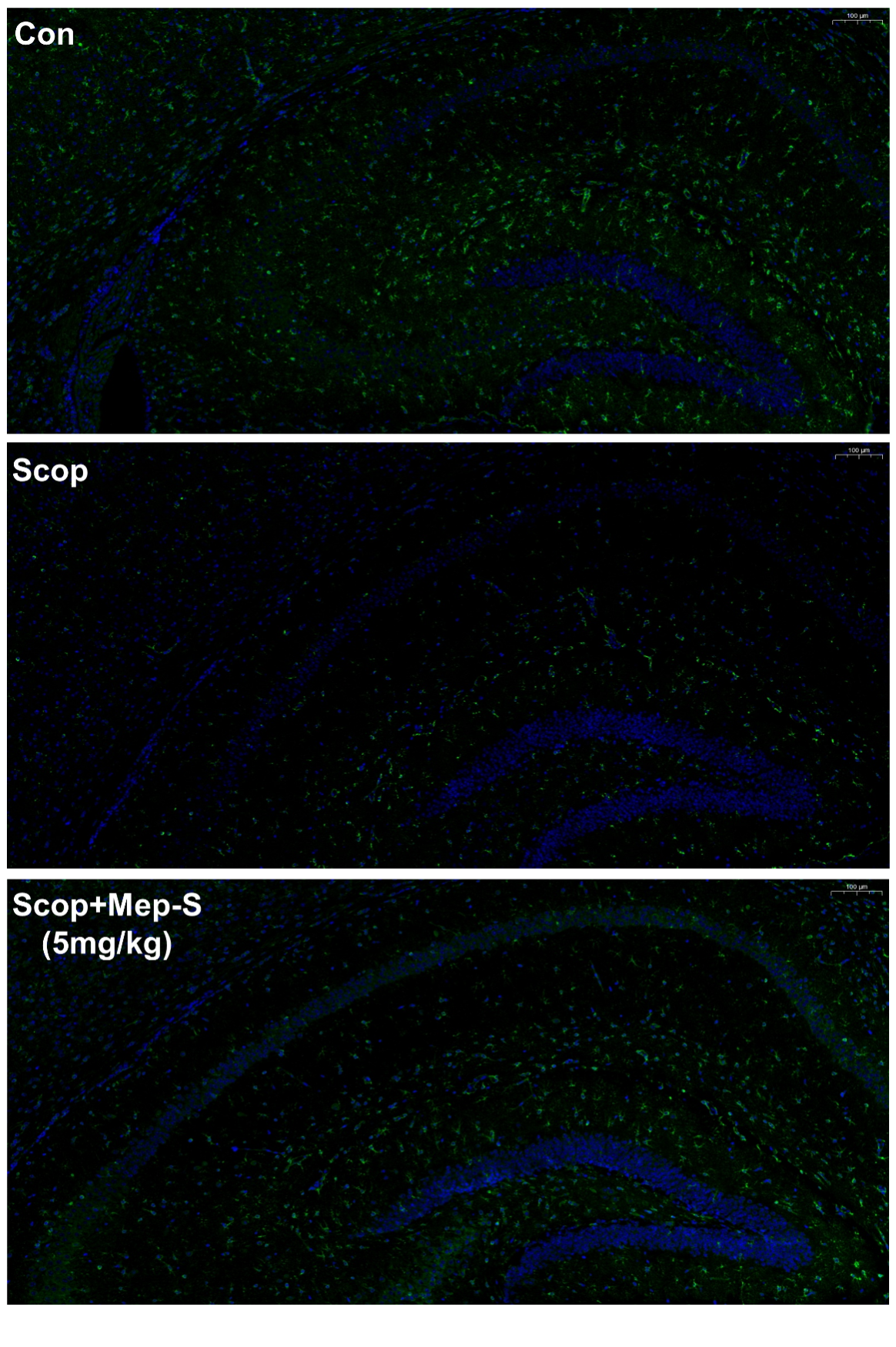


**Figure S3**. Representativephotographs of immunofluorescence staining of Nrf2 (green) and DAPI (blue) in mousehippocampus of the control, scopolamine-treated, and scopolamine+Mep-S-treated groups.Magnification, ×200.
